# Moderate Restriction of Macrophage-Tropic Human Immunodeficiency Virus Type 1 by SAMHD1 in Monocyte-Derived Macrophages

**DOI:** 10.1371/journal.pone.0090969

**Published:** 2014-03-05

**Authors:** Kahoru Taya, Emi E. Nakayama, Tatsuo Shioda

**Affiliations:** Department of Viral Infections, Research Institute for Microbial Diseases, Osaka University, Suita, Osaka, Japan; Lady Davis Institute for Medical Research, Canada

## Abstract

Macrophage-tropic human immunodeficiency virus type 1 (HIV-1) strains are able to grow to high titers in human monocyte-derived macrophages. However, it was recently reported that cellular protein SAMHD1 restricts HIV-1 replication in human cells of the myeloid lineage, including monocyte-derived macrophages. Here we show that degradation of SAMHD1 in monocyte-derived macrophages was associated with moderately enhanced growth of the macrophage-tropic HIV-1 strain. SAMHD1 degradation was induced by treating target macrophages with vesicular stomatitis virus glycoprotein-pseudotyped human immunodeficiency virus type 2 (HIV-2) particles containing viral protein X. For undifferentiated monocytes, HIV-2 particle treatment allowed undifferentiated monocytes to be fully permissive for productive infection by the macrophage-tropic HIV-1 strain. In contrast, untreated monocytes were totally resistant to HIV-1 replication. These results indicated that SAMHD1 moderately restricts even a macrophage-tropic HIV-1 strain in monocyte-derived macrophages, whereas the protein potently restricts HIV-1 replication in undifferentiated monocytes.

## Introduction

CD4 is the primary receptor molecule of human immunodeficiency virus type 1 (HIV-1) for viral attachment to the target cells [1]. HIV-1 thus replicates in CD4^+^ cells such as activated human CD4^+^ lymphocytes and macrophages [2], [3]. Among different HIV-1 strains, macrophage-tropic HIV-1 strains replicate particularly well in cultured human monocyte-derived macrophages [4]. Many macrophage-tropic HIV-1 strains fail to replicate well in established human T cell lines such as Hut78 and MT4, cell lines in which laboratory-adapted T-cell line-tropic HIV-1 strains can replicate efficiently. Conversely, many laboratory-adapted T-cell line-tropic HIV-1 strains fail to replicate well in monocyte-derived macrophages [5]. Sequence variations in the HIV-1 envelope protein, especially in the third variable region, correlate with the HIV-1 cellular host range [6]–[9], and this observation led to the identification of the CCR5 and CXCR4 chemokine receptors as HIV-1 co-receptors for viral fusion with target cell membranes [10]–[16]. Macrophage-tropic HIV-1 strains utilize CCR5 as a co-receptor, and most such macrophage-tropic HIV-1 strains now have been re-designated as R5-tropic strains, although not all the R5-tropic HIV-1 strains can efficiently replicate in macrophages [17]–[19]. Laboratory-adapted T-cell line-tropic HIV-1 strains utilize CXCR4 as a co-receptor, and most such T-cell line-tropic HIV-1 strains now have been re-designated as X4-tropic strains, although CXCR4 expression also was observed in macrophages [20]–[22], cells in which X4-tropic strains cannot replicate well.

Despite the presence of the aforementioned HIV-1 strains that can replicate well in macrophages, it has been also reported that HIV-1-based lentivirus vectors composed of HIV-1 Gag and Pol proteins and vesicular stomatitis virus glycoprotein (VSV-G) showed markedly reduced efficiency for transduction of cells of myeloid lineage [23], [24]. The restriction was rather strong in monocyte-derived dendritic cells and to a lesser extent in monocyte-derived macrophages [25], [26]. Such a myeloid lineage-specific restriction was not observed in lentivirus vectors based on simian immunodeficiency virus isolated from macaques (SIVmac) [26], which is in the same lineage as human immunodeficiency virus type 2 (HIV-2), or in simian immunodeficiency virus isolated from sooty mangabey (SIVsm). The myeloid lineage-specific restriction of HIV-1-based lentivirus vector could also be abrogated by pretreatment of cells with SIVmac particles [27]. Members of HIV-2 and SIVsm lineage encode a non-structural viral protein X (Vpx) that is absent from HIV-1. VpX was shown to abrogate the myeloid lineage-specific restriction of HIV-1-basesd lentivirus vectors [27]–[29].

In 2011, SAMHD1 (a cellular protein SAM- and HD-domain-containing protein) was implicated as a target of Vpx that was responsible for abrogation of HIV-1 restriction in human cells of myeloid lineage [30], [31]. Subsequently, SAMHD1 was shown to restrict HIV-1 infection in resting CD4^+^ T cells [32]. SAMHD1 possesses deoxynucleoside triphosphate triphosphohydrolase activity; this activity reduces levels of deoxynucleoside triphosphate in cells of myeloid lineage and resting CD4^+^ cells, thereby preventing reverse-transcription of HIV-1 RNA in these cell types [33], [34]. Vpx antagonizes SAMHD1 and induces proteolytic degradation of SAMHD1 through the CUL4A/DCAF1 E3 ubiquitin ligase complex [30].

In most of the SAMHD1 studies cited above, the efficiency of HIV-1 infection was assayed in the context of lentivirus vectors composed of HIV-1 Gag and Pol proteins packaged with VSV-G protein, along with reporter genes such as those encoding luciferase or green fluorescent protein. This distinction raises the question of whether SAMHD1 provides the same function in live HIV-1 viruses. Therefore, in the study presented here, we re-evaluated the role of SAMHD1 in HIV-1 replication in monocyte-derived macrophages in the context of a live macrophage-tropic HIV-1 strain that can replicate well in macrophages.

## Results

### Lack of Enhancing Effect of Macrophage-tropic HIV-1 Strain on HIV-1 Infection in Monocyte-derived Macrophages

**Figure 1:**
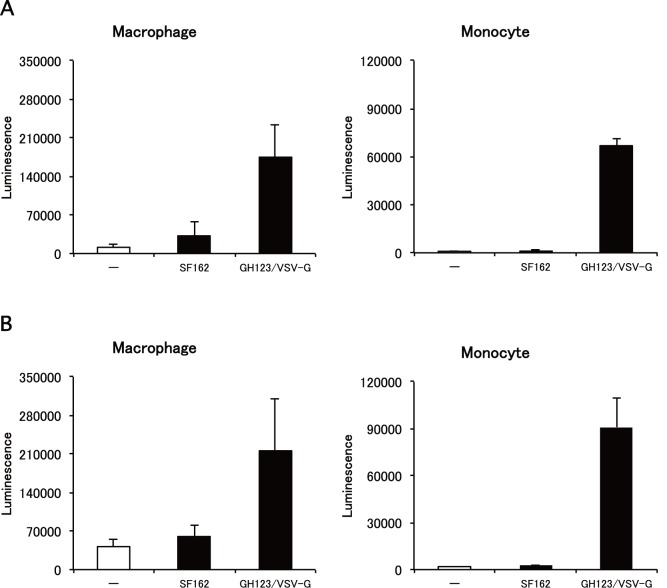
Effects of macrophage-tropic HIV-1 strain and VSV-G-pseudotyped HIV-2 particles on lentivirus vector infection. Monocytes were differentiated into macrophages for 11 days in the presence of GM-CSF. Macrophages or monocytes were treated with macrophage-tropic HIV-1 strain (SF162) or VSV-G pseudotyped and Env-defective HIV-2 particles (GH123/VSV-G) and then infected with lentivirus vector NL43-Luci/VSV-G. Luciferase activity was measured 4 days after infection. Data are plotted as the mean ± SD of triplicate samples; presented data are representative of three independent experiments using two donors. A: Results of samples obtained from a donor 1. B: Results of samples obtained from a donor 2.

SAMHD1 was reported to suppress infection of HIV-1-based lentivirus vectors containing VSV-G in cultured monocyte-derived macrophages [30]. On the other hand, macrophage-tropic HIV-1 strains can efficiently replicate in monocyte-derived macrophages [4]. To reconcile these potentially contradictory results, we tested the hypothesis that live macrophage-tropic HIV-1 strains can evade restriction of SAMHD1 by an unidentified mechanism. As a first step, we treated monocyte-derived macrophages with a macrophage-tropic HIV-1 strain SF162 before inoculation with VSV-G-pseudotyped lentivirus vector expressing luciferase (NL43-Luci/VSV-G). Results showed that pretreatment with SF162 failed to enhance subsequent infection by NL43-Luci/VSV-G in macrophages (Fig. 1A and 1B, left panels), whereas pretreatment with VSV-G-pseudotyped and Env-defective HIV-2 particles containing Vpx (GH123-Nhe/VSV-G) enhanced luciferase expression (Fig. 1A and 1B, left panels), as reported previously [30]. The effect of GH123-Nhe/VSV-G was more prominent in undifferentiated monocytes than in fully differentiated macrophages; a more than 200-fold increase of luciferase activity was observed by pretreatment of monocytes with GH123-Nhe/VSV-G (Fig. 1A and 1B, right panels). SF162 again failed to enhance subsequent infection by NL43-Luci/VSV-G in monocytes (Fig. 1A and 1B, right panels).

### Enhancing Effect of VSV-G Pseudotyped HIV-2 Particles on Macrophage-tropic HIV-1 Strain in Monocyte-derived Macrophages

**Figure 2:**
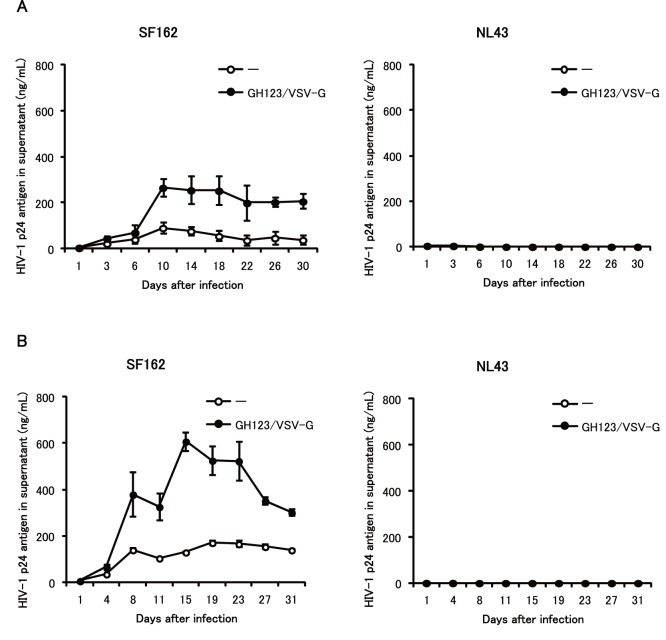
Effects of VSV-G-pseudotyped HIV-2 particles on macrophage-tropic and T-cell line-tropic HIV-1 strains in GM-CSF-induced macrophages. Monocytes were differentiated into macrophages for 6 days in the presence of GM-CSF. Macrophages were treated with VSV-G pseudotyped and Env-defective HIV-2 particles (GH123/VSV-G) and then infected with HIV-1 strain SF162 or NL43. HIV-1 replication was quantified by ELISA measurement of p24 antigen in the supernatant after infection. Data are plotted as the mean ± SD of triplicate samples; presented data are representative of three independent experiments using two donors. A: Results of samples obtained from a donor 1. B: Results of samples obtained from a donor 2.

We next tested whether GH123-Nhe/VSV-G also could enhance replication of a macrophage-tropic HIV-1 strain in monocyte-derived macrophages. Fig. 2 shows the effects of GH123-Nhe/VSV-G on replication of SF162 in cultured macrophages that were differentiated from monocytes with granulocyte-macrophage colony stimulating factor (GM-CSF). SF162 replicated to titers corresponding to approximately 100 ng/ml of p24 core protein (Fig. 2A and 2B, left panels). Up to five-fold higher titers were detected in SF162-infected macrophages pretreated with GH123-Nhe/VSV-G (Fig. 2A and 2B, left panels). These results indicated that GH123-Nhe/VSV-G could enhance replication of macrophage-tropic HIV-1 in GM-CSF-differentiated macrophage cultures. In contrast, the T-cell line-tropic HIV-1 strain NL43 did not replicate at all in macrophages, regardless of the presence or absence of GH123-Nhe/VSV-G treatment (Fig. 2A and B, right panels).

**Figure 3:**
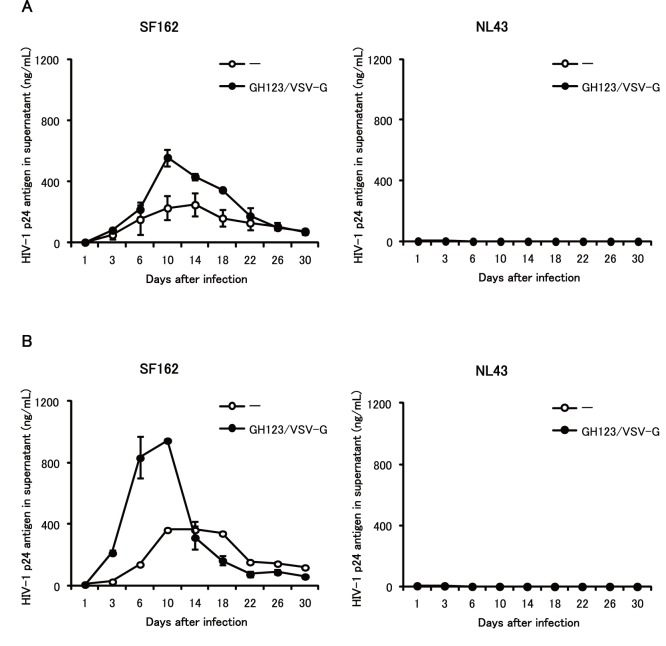
Effects of VSV-G-pseudotyped HIV-2 particles on macrophage-tropic and T-cell line-tropic HIV-1 strains in M-CSF-induced macrophages. Monocytes were differentiated into macrophages for 6 days in the presence of M-CSF. Macrophages were treated with VSV-G pseudotyped and Env-defective HIV-2 particles (GH123/VSV-G) and then infected with HIV-1 strain SF162 or NL43. HIV-1 replication was quantified by ELISA measurement of p24 antigen in the supernatant after infection. Data are plotted as the mean ± SD of triplicate samples; presented data are representative of two independent experiments using two donors. A: Results of samples obtained from a donor 1. B: Results of samples obtained from a donor 2.

Monocyte-derived macrophages differentiated with macrophage colony stimulating factor (M-CSF) are more susceptible to HIV-1 infection than those differentiated with GM-CSF [35]–[38]. We therefore tested whether GH123-Nhe/VSV-G could enhance SF162 replication in monocyte-derived macrophages differentiated with M-CSF. SF162 grew to titers corresponding to 200 ng/ml of p24 in donor 1 macrophages (Fig. 3A, left) and to 400 ng/ml of p24 in donor 2 macrophages (Fig. 3B, left), a level at least two-fold higher than those found in SF162-infected macrophage cultures differentiated with GM-CSF. Up to five-fold higher titers of SF162 also were detected in M-CSF-differentiated macrophage cultures pretreated with GH123-Nhe/VSV-G (Fig. 3A and 3B, left panels). These results indicated that GH123-Nhe/VSV-G could enhance replication of macrophage-tropic HIV-1 replication even in M-CSF-differentiated macrophages. The T-cell line-tropic NL43 strain again did not replicate at all in M-CSF-differentiated macrophages, regardless of the presence or absence of GH123-Nhe/VSV-G treatment (Fig. 3A and 3B, right panels).

### Enhancing Effect of VSV-G-pseudotyped HIV-2 Particles on Macrophage-tropic HIV-1 Strain in Undifferentiated Monocytes

**Figure 4:**
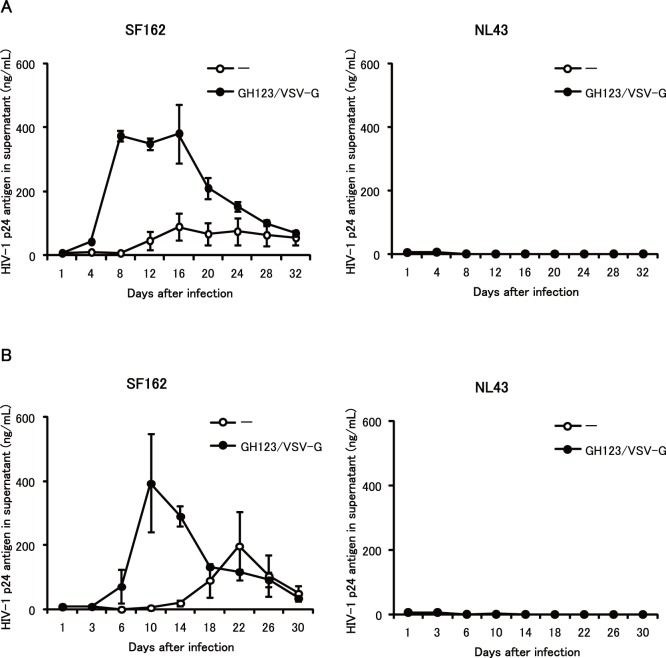
Effects of VSV-G-pseudotyped HIV-2 particles on macrophage-tropic and T-cell line-tropic HIV-1 strains in undifferentiated monocytes. Monocytes were treated with VSV-G pseudotyped and Env-defective HIV-2 particles (GH123/VSV-G) and then infected with HIV-1 strain SF162 or NL43. HIV-1 replication was quantified by ELISA measurement of p24 antigen in the supernatant after infection. Data are plotted as the mean ± SD of triplicate samples obtained from a single blood donor; presented data are representative of three independent experiments using two donors. A: Results of samples obtained from a donor 1. B: Results of samples obtained from a donor 2.

**Figure 5:**
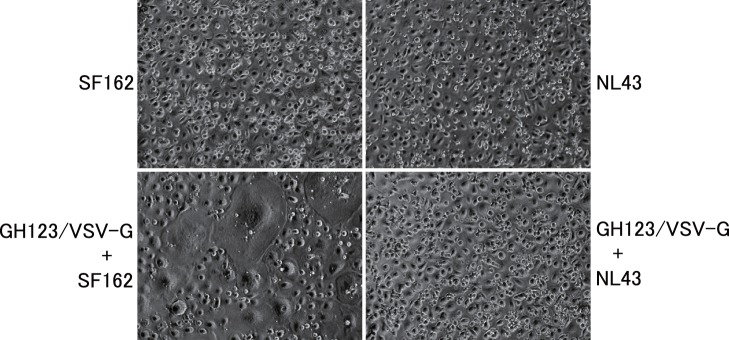
Syncytium formation in undifferentiated monocytes pretreated with HIV-2 particles and then infected with macrophage-tropic HIV-1. Monocytes were treated with VSV-G pseudotyped and Env-defective HIV-2 particles (GH123/VSV-G) and then infected with HIV-1 strain SF162 or NL43. Presented data are representative of two independent experiments.

In contrast to differentiated macrophages, undifferentiated monocytes are highly resistant to HIV-1 infection, but treatment with GH123-Nhe/VSV-G greatly enhanced VSV-G-pseudotyped lentivirus vector transduction (Fig. 1A and 1B, right panels). This pattern also was observed for live SF162 infection of undifferentiated monocytes (Fig. 4 and 5). SF162 replication in monocytes was not observed until 12 days after infection, when cell morphology suggested that some of the monocytes in the culture had differentiated into macrophages. On the other hand, GH123-Nhe/VSV-G rendered cultured monocytes fully permissive for SF162 replication (Fig. 4A and 4B, left panels). Up to 50-fold higher titers of SF162 were detected in monocytes treated with GH123-Nhe/VSV-G than in untreated monocytes. Consistently, large multi-nucleated syncytia were observed 6 days after infection, but only in monocytes treated with GH123-Nhe/VSV-G (Fig. 5). These results are in good agreement with recent findings that non-stimulated CD14^+^ cells obtained from Aicardi-Goutieres syndrome patients (who are homozygous for a nonsense mutation in the SAMHD1-encoding gene) were highly susceptible to macrophage-tropic HIV-1 infection [39]. As with GM-CSF- and M-CSF-differentiated macrophages, the T-cell line-tropic NL43 strain did not replicate at all in undifferentiated monocytes, regardless of the presence or absence of GH123-Nhe/VSV-G treatment (Fig. 4A and 4B, right panels).

### Levels of SAMHD1 Expression in Monocytes and Macrophages

**Figure 6:**
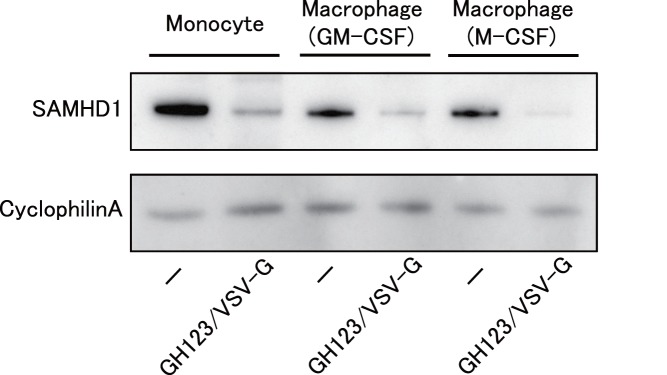
Western blot analysis of SAMHD1 in undifferentiated monocytes and macrophages. Monocytes were differentiated into macrophages for 6 days in the presence of GM-CSF or M-CSF. Macrophages or monocytes were treated with or without VSV-G-pseudotyped and Env-defective HIV-2 particles (GH123/VSV-G), and harvested. Whole-cell extracts were separated on SDS-PAGE and analyzed by western blot using the indicated antibodies. Presented data are representative of two independent experiments.

Vpx was reported to induce proteolytic degradation of SAMHD1. We therefore compared levels of SAMHD1 protein expression in cells treated with GH123-Nhe/VSV-G and those without treatment. As shown in Fig. 6, GH123-Nhe/VSV-G markedly reduced levels of SAMHD1 protein expression in monocytes and macrophages. Consistent with the previous observation [31], levels of SAMHD1 protein expression were apparently higher in undifferentiated monocytes than in macrophages.

**Figure 7:**
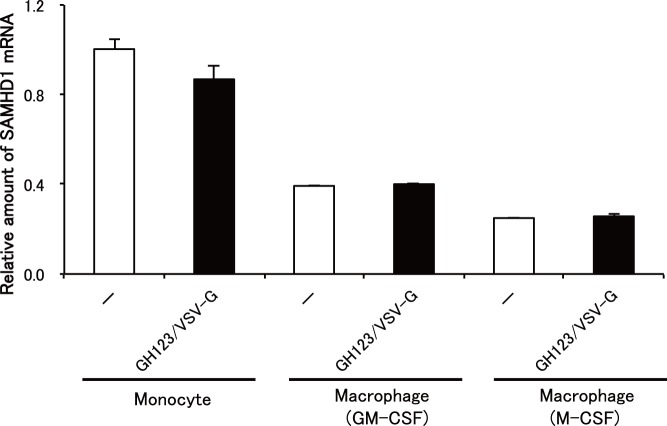
Quantification of SAMHD1 mRNA expression. Monocytes were differentiated into macrophages for 6 days in the presence of GM-CSF or M-CSF. Macrophages or monocytes were treated with or without VSV-G-pseudotyped and Env-defective HIV-2 particles (GH123/VSV-G), and harvested. SAMHD1 mRNA expression levels were detected by real-time RT-PCR and normalized against GAPDH. Data are shown as mean ± SD.

We then quantitated levels of SAMHD1 mRNA expression in monocytes and macrophages. As expected, SAMHD1 mRNA levels were higher in monocytes than in macrophages (Fig. 7). There was no difference in SAMHD1 mRNA levels between cells treated with GH123-Nhe/VSV-G and those without treatment. These results confirmed that reduced SAMHD1 protein levels in cells treated with GH123-Nhe/VSV-G were caused by enhanced degradation of SAMHD1 by HIV-2 particles containing Vpx.

### Phosphorylation of SAMHD1 in Monocytes and Differentiated Macrophages

**Figure 8:**
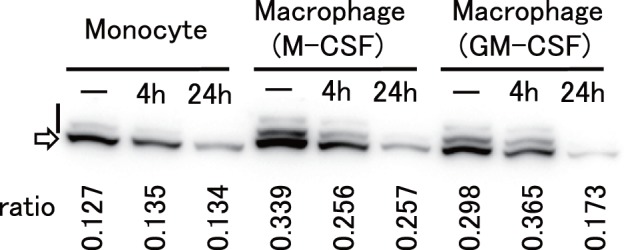
Phosphorylation state of SAMHD1. Monocytes (Monocyte), M-CSF-differentiated macrophages (Macrophage (M-CSF)), and GM-CSF-differentiated macrophages (Macrophage (GM-CSF)) were treated with GH123-Nhe/VSV-G for 2 h and incubated at 37°C for 4 h (4 h) or 24 h (24 h), or mock-treated (−). Cells were lyzed and subjected for SDS-PAGE containing Phos-tag to separate phosphorylated proteins from nonphosphorylated ones. SAMHD1 proteins were detected by anti-SAMHD1 antibody. Upper bands shown by a vertical bar and a lower band shown by an arrow represent phosphorylated and nonphosphorylated SAMHD1, respectively. Ratios of phosphorylated SAMHD1 levels to total SAMHD1 levels (ratio) are shown in vertical numbers.

It was recently reported that Cyclin A2/CDK1 phosphorylates SAMHD1 at the threonine 592 residues. Phosphorylation of the SAMHD1 threonine 592 correlates with loss of its ability to restrict HIV-1 [40]–[42]. We therefore analyzed the phosphorylation state of SAMHD1 in monocytes and differentiated macrophages. As shown in Fig. 8, SAMHD1 proteins in monocytes were less phosphorylated than those in GM-CSF-differentiated or M-CSF-differentiated macrophages. This result is in good agreement with those reported previously [40], and correlated well with our results that SAMHD1 restriction in monocytes was much more potent than that in differentiated macrophages (Fig. 1 and 4). Treatment with GH123-Nhe/VSV-G reduced both phosphorylated and nonphosphorylated SAMHD1 in all cells. There was no difference in the phosphorylation state of SAMHD1 between GM-CSF-differentiated and M-CSF-differentiated macrophages (Fig. 8).

### Enhancing Effect of SAMHD1 siRNA on HIV-1 Infection in Monocytes and Monocyte-derived Macrophages

**Figure 9:**
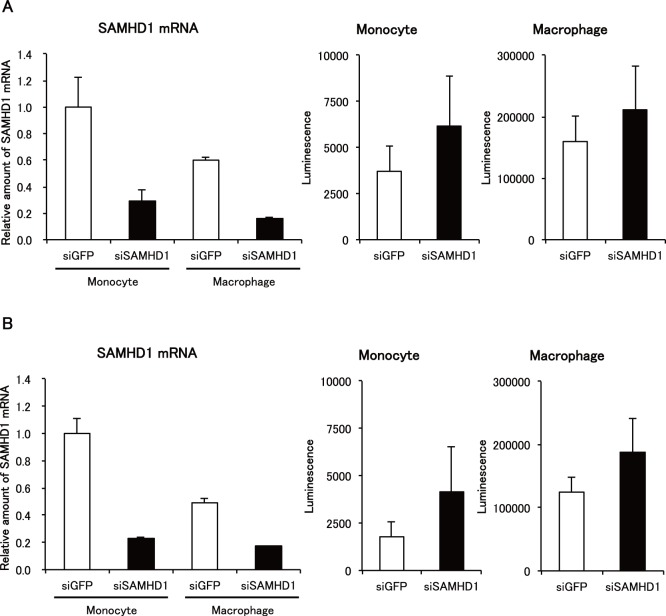
Effects of SAMHD1 siRNA on HIV-1 infection. Monocytes (Monocyte) or macrophages (Macrophage) were treated with SAMHD1- (siSAMHD1) or GFP- (siGFP) targeting siRNAs. Three days after transfection, cells were harvested or infected with NL43-Luci/VSV-G virus. SAMHD1 mRNA expression levels in harvested cells were detected by real-time RT-PCR and normalized against GAPDH. Data are shown as mean ± SD. Luciferase activity in infected cells was measured 4 days after infection. Data are plotted as the mean ± SD of triplicate samples; presented data are representative of two independent experiments using two donors. A: Results of samples obtained from a donor 1. B: Results of samples obtained from a donor 2.

To confirm that the observed enhancing effect of VSV-G-pseudotyped HIV-2 particles on HIV-1 infection was due to degradation of SAMHD1 in monocytes and macrophages, we used siRNAs targeting SAMHD1 to reduce levels of SAMHD1 expression. The siRNAs targeting SAMHD1 reduced levels of SAMHD1 mRNA in both monocytes and GM-CSF-differentiated macrophages (Fig. 9A and 9B, left panels). Accordingly, increased levels of NL43-Luci/VSV-G infection were observed in monocytes (Fig. 9A and 9B, middle panels) and GM-CSF-differentiated macrophages (Fig. 9A and 9B, right panels) transfected with the siRNAs targeting SAMHD1. These results confirmed that the enhancing effect of VSV-G-pseudotyped HIV-2 particles on HIV-1 infection was due to degradation of SAMHD1.

## Discussion

SAMHD1 was reported to suppress HIV-1 infection of cells of myeloid lineage, including monocyte-derived macrophages [30], [31]. Nevertheless, macrophage-tropic HIV-1 strains can efficiently replicate in monocyte-derived macrophages [4], [6]–[8]. In the present study, we have shown that the treatment of monocyte-derived macrophages or undifferentiated monocytes with GH123-Nhe/VSV-G enhanced subsequent macrophage-tropic HIV-1 infection. GH123-Nhe/VSV-G treatment reduced levels of SAMHD1 protein expression in monocyte-derived macrophages and undifferentiated monocytes. Enhancing effects by GH123-Nhe/VSV-G were observed even in M-CSF-induced monocyte-derived macrophages, which were reported to be highly susceptible to HIV-1 infection [35]–[38], [43]. These results indicated that SAMHD1 could moderately suppress replication of the macrophage-tropic HIV-1 strain in monocyte-derived macrophages. It is formally possible that live macrophage-tropic HIV-1 strains evade restriction of SAMHD1 by an unidentified mechanism, although we consider such a possibility unlikely.

Levels of HIV-1 restriction by SAMHD1 in monocyte-derived macrophages were rather modest, while those in undifferentiated monocytes were quite strong (Fig. 2, 3 and 4). SAMHD1 expression levels were higher in undifferentiated monocytes than in monocyte-derived macrophages (Fig. 6 and 7), showing a clear correlation between levels of SAMHD1 expression and those of restriction. Furthermore, phosphorylation of SAMHD1, which was reported to abolish HIV-1 restriction activity of SAMHD1 [40]–[42], was more prominent in M-CSF-induced or GM-CSF-induced macrophages than in undifferentiated monocytes (Fig. 8). When we compared M-CSF-induced and GM-CSF-induced macrophages, HIV-1 grew to higher titers in M-CSF-induced macrophages than in GM-CSF-induced macrophages. This result is in good agreement with those of the previous studies [37], [43]. The levels of restriction by SAMHD1 also were slightly lower in M-CSF-induced macrophages than in GM-CSF-induced macrophages, although we failed to observe clear differences in levels of expression or phosphorylation state of SAMHD1 between M-CSF-induced and GM-CSF-induced macrophages (Fig. 6, 7, and 8). Thus, mechanisms underlying the higher sensitivity to HIV-1 infection in M-CSF-induced macrophages are not clear at present; further studies are necessary, including comparisons of CD4 and CCR5 expression levels between M-CSF-induced and GM-CSF-induced macrophages.

It should be noted here that the effects of GH123-Nhe/VSV-G treatment seem to last relatively long, although we treated cells only once with GH123-Nhe/VSV-G. It is possible that the Vpx protein produced by the transduced HIV-2 genomes was incorporated into infectious SF162 progeny virions and thus facilitated replication of SF162 in the next cycle of infection. It is also possible that Env-deficient progeny HIV-2 virions were pseudotyped with fully functional SF162 envelope proteins, and thereby continued to produce Vpx that degraded SAMHD1.

Similar to monocytes and macrophages, microglial cells in the human brain are derived via the myeloid lineage and are also susceptible to HIV-1 infection [44]. HIV-1-infected microglial cells appear to play an important role in HIV-1-associated neurological disorders such as dementia and neurocognitive disorder [45]. It therefore would be interesting to investigate whether or not SAMHD1 moderately suppresses HIV-1 replication in microglial cells just as in macrophages. If SAMHD1 can suppress HIV-1 replication at least moderately in microglial cells, artificial potentiation of SAMHD1 in microglial cells might be a novel approach to the treatment of HIV-1-associated neurological disorders.

In conclusion, we have shown that SAMHD1 moderately restricts even macrophage-tropic HIV-1 strains in monocyte-derived macrophages. SAMHD1 restriction was much more potent in undifferentiated monocytes than that in GM-CSF-differentiated or M-CSF-differentiated macrophages. Levels of expression and phosphorylation state of SAMHD1 could at least partially explain the different levels of SAMHD1 restriction between undifferentiated monocytes and differentiated macrophages.

## Materials and Methods

### Ethics Statement

Peripheral blood mononuclear cells (PBMC) were obtained from healthy donors with written informed consent. Use of human materials in this study was approved by the Research Ethics Committee of Osaka University.

### Viruses

Macrophage-tropic HIV-1 strain SF162 and laboratory-adapted T-cell line-tropic HIV-1 strain NL43 were grown in CCR5-expressing MT4 cells [46] and titrated for use with the RETROtek Antigen ELISA kit (ZeptoMetrix, Buffalo, NY). VSV-G-pseudotyped lentivirus vector expressing luciferase (NL43-Luci/VSV-G) and VSV-G-pseudotyped and Env-defective HIV-2 particles containing Vpx (GH123-Nhe/VSV-G) were produced from human embryonic kidney cells (293T cells) using polyethylenimine (PEI) (molecular weight, 25,000; Polysciences, Warrington, PA). Briefly, for NL43-Luci/VSV-G virus production, 293T cells were transfected with 15 µg of pNL4-3-Luc-R-E- plasmid and 5 µg of VSV-G-encoding plasmid; for GH123-Nhe/VSV-G virus production, 293T cells were transfected with 15 µg of GH123-Nhe and 5 µg of VSV-G-encoding plasmid. The GH123-Nhe plasmid was generated by blunting the *Nhe*I site in the HIV-2 GH123 plasmid, thereby introducing a frame-shift mutation in its *env* gene. For transfected cells, medium was replaced 6 h after transfection, and viruses were harvested 48 h later. Viral titers were measured with the RETROtek Antigen ELISA kit.

### Cells

PBMCs were obtained from blood buffy coats of healthy donors using Ficoll-Paque density gradient centrifugation, and then plated in 24-well, 12-well, or 6-well MULTIWELL PRIMARIA plates (Becton Dickinson, Franklin Lakes, NJ) with RPMI 1640 supplemented with 10% fetal calf serum (FCS). To obtain the monocyte population, the floating cells were removed by washing the plates with phosphate-buffered saline (PBS) four times after incubation at 37°C for 1 day. Monocytes were differentiated into macrophages for 6–11 days in the presence of 100 ng/ml of granulocyte macrophage colony stimulating factor (GM-CSF) (PeproTECH, Rocky Hill, NJ) or 100 ng/ml of macrophage colony stimulating factor (M-CSF) (PeproTECH).

### Luciferase Assay

Macrophages (1.6–2.4×10^5^ cells) and monocytes (2.6–3.4×10^6^ cells) were pretreated for 2 h with a titer of macrophage-tropic HIV-1 strain SF162 virus equivalent to 100 ng of p24 or with a titer of GH123-Nhe/VSV-G virus equivalent to 100 ng of p27, and then infected with a titer of NL43-Luci/VSV-G virus equivalent to 7.7 ng of p24. After incubation for 2 h, the medium was changed, and cells were incubated for 4 days at 37°C. Luciferase activity was measured in cell lysates according to the manufacturer’s instructions (Bright-Glo Luciferase Assay System, Promega, Madison, WI) and read using a Centro LB960 Microplate Luminometer (Berthold, Bad Wildbad, Germany).

### Virus Infection

Macrophages and monocytes (2.2–2.6×10^6^ cells) were pretreated for 2 h with a titer of GH123-Nhe/VSV-G virus equivalent to 100 ng of p27, and then infected with a titer of virus equivalent to 100 ng of p24 of macrophage-tropic HIV-1 strain SF162 or laboratory-adapted T-cell line-tropic HIV-1 strain NL43. After incubation for 2 h, cells were washed with PBS and incubated with RPMI 1640 supplemented with 10% FCS and 100 ng/ml GM-CSF or 100 ng/ml M-CSF. Culture supernatants were collected periodically, and levels of HIV-1 p24 antigen were measured by the RETROtek HIV-1 p24 Antigen ELISA kit.

### Western Blot

Monocytes and macrophages (3.5×10^6^ cells) were lysed in Laemmli sample buffer (100 mM Tris-HCl, pH 6.8, 0.04% sodium dodecyl sulfate (SDS), 20% glycerol, 0.12% 2-mercaptoethanol). Proteins in the lysates were subjected to SDS-polyacrylamide gel electrophoresis (SDS-PAGE). Proteins in the gel were then electrically transferred to a membrane (Immobilion; Millipore, Billerica, MA). Blots were blocked and probed with anti-CypA affinity rabbit polyclonal antibody (Sigma, St. Louis, MO) overnight at 4°C. Blots then were incubated with peroxidase-linked protein A (GE Healthcare, Buckinghamshire, UK), and bound antibodies were visualized with a Chemilumi-One chemiluminescent kit (Nacalai Tesque, Kyoto, Japan). Quantities of cell lysate were normalized by CypA level, then subjected to a new round of SDS-PAGE and membrane transfer. For the new blot, SAMHD1 protein in the membrane was detected with anti-SAMHD1 (611–625) rabbit antibody (Sigma) followed by peroxidase-linked protein A (GE Healthcare, Buckinghamshire, UK) detection as described above.

### Phosphorylation State of SAMHD1

Monocyte or macrophages (6×10^6^ cells) were treated with a titer of GH123-Nhe/VSV-G virus equivalent to 500 ng of p25 for 2 h, washed with medium, and then incubated at 37°C for 4 h or 24 h. Cells were lysed in Laemmli sample buffer. Proteins in the lysates were separated by SDS-PAGE containing Phos-tag (Wako, Osaka, Japan), a ligand that decreases the mobility of phosphorylated proteins. Separated proteins in the gel were analyzed by western blotting using anti-SAMHD1 antibody. The gel images were analyzed by CS analyzer 3.0 (ATTO, Tokyo, Japan).

### Quantification of SAMHD1 mRNA Expression

Total RNA was extracted from monocytes or macrophages using TRIZOL reagent (Life Technologies, Carlsbad, CA) following the manufacturer’s instructions. cDNA was synthesized from 1 µg of total RNA using the High-capacity cDNA Archive kit (Applied Biosystems, Carlsbad, CA) according to the manufacturer’s instructions. For real time PCR, each 20-µL reaction mixture consisted of 5 µL of cDNA, 10 µL TaqMan Universal PCR Master Mix (Applied Biosystems, Carlsbad, CA) and 1 µL of TaqMan Gene Ex Assays (Assay ID: Hs00210019_m1). Real time PCR was performed with an Applied Biosystems 7500 Real-Time PCR System. Levels of SAMHD1 mRNA were normalized with those of GAPDH according to the manufacturer’s instructions.

### RNA Interference

The cultured medium of monocytes (1.6–2.4×10^6^ cells) was replaced with 0.5 mL of Opti-MEM (Gibco, Carlsbad, CA) before transfection. The SAMHD1-targeting pool comprised of the siRNAs to the following target sequences: J-013950-09: 5′-GACAAUGAGUUGCGUAUUU-3′; J-013950-10: 5′-CAUGUUUGAUGGACGAUUU-3′; J-013950-11: 5′-AAGUAUUGCUAGACGUGAA-3′; J-013950-12: 5′-UUAGUUAUAUCCAGCGAUU-3′ were purchased from Dharmacon (Lafayette, CO). The GFP-targeting siRNA to the sequence 5′-GGCTACGTCCAGGAGCGCACC-3′ were used as a control siRNA. Monocytes were transfected with 40 pmol aliquots of siRNA with 2 µL of Lipofectamine 2000 (Invitrogen, Carlsbad, CA) per well. Six h after transfection, cultured medium was replaced with RPMI 1640 supplemented with 10% FCS. At day 3, monocytes were infected with a titer of NL43-Luci/VSV-G virus equivalent to 7.7 ng of p24. In the case of GM-CSF-induced macrophages (1.6–3.6×10^6^ cells), siRNAs were transfected at day 4 after differentiation by GM-CSF, and then infected with NL43-Luci/VSV-G virus at day 7. Total RNA was extracted from cells at the time point of NL43-Luci/VSV-G virus infection for quantification of SAMHD1 mRNA expression.
